# Screening and Testing for Hepatitis B Virus Infection: CDC Recommendations — United States, 2023

**DOI:** 10.15585/mmwr.rr7201a1

**Published:** 2023-03-10

**Authors:** Erin E. Conners, Lakshmi Panagiotakopoulos, Megan G. Hofmeister, Philip R. Spradling, Liesl M. Hagan, Aaron M. Harris, Jessica S. Rogers-Brown, Carolyn Wester, Noele P. Nelson, Contributors, Karina Rapposelli, Amy L. Sandul, Elisa Choi, Carla Coffin, Kristen Marks, David L. Thomas, Su H. Wang

**Affiliations:** 1 Division of Viral Hepatitis, National Center for HIV, Viral Hepatitis, STD, and TB Prevention, CDC; Division of Viral Hepatitis, National Center for HIV, Viral Hepatitis, STD, and TB Prevention,; CDC; Division of Viral Hepatitis, National Center for HIV, Viral Hepatitis, STD, and TB Prevention, CDC; Harvard Vanguard Medical Associates Somerville and American College of Physicians; Calvin, Phoebe and Joan Snyder Institute for Chronic Diseases, Cumming School of Medicine, University of Calgary and Calgary Liver Unit, Calgary Division of Gastroenterology and Hepatology, Alberta Health Services; Division of Infectious Disease, Weill Cornell Medical College; Johns Hopkins School of Medicine and Bloomberg School of Public Health; Cooperman Barnabas Medical Center and RWJ Barnabas-Rutgers Medical Group.

## Abstract

*Chronic hepatitis B virus (HBV) infection can lead to substantial morbidity and mortality. Although treatment is not considered curative, antiviral treatment, monitoring, and liver cancer surveillance can reduce morbidity and mortality. Effective vaccines to prevent hepatitis B are available. This report updates and expands CDC’s previously published* Recommendations for Identification and Public Health Management of Persons with Chronic Hepatitis B Virus Infection (MMWR Recomm Rep 2008;57[No. RR-8]) *regarding screening for HBV infection in the United States*. *New recommendations include hepatitis B screening using three laboratory tests at least once during a lifetime for adults aged* ≥*18 years. The report also expands risk-based testing recommendations to include the following populations, activities, exposures, or conditions associated with increased risk for HBV infection: persons incarcerated or formerly incarcerated in a jail, prison, or other detention setting; persons with a history of sexually transmitted infections or multiple sex partners; and persons with a history of hepatitis C virus infection. In addition, to provide increased access to testing, anyone who requests HBV testing should receive it, regardless of disclosure of risk, because many persons might be reluctant to disclose stigmatizing risks.*

## Introduction

Persons with chronic hepatitis B virus (HBV) infection are at increased risk for liver cancer and cirrhosis and are 70%–85% more likely to die prematurely than the general population (*1*–*4*). An estimated 580,000 to 2.4 million persons are living with HBV infection in the United States (*5*,*6*), two thirds of whom might be unaware of their infection (*5*). Chronic HBV infection disproportionately affects persons born outside the United States; non-U.S.–born persons account for 14% of the general population, but account for 69% of the U.S. population living with chronic HBV infection (*5*–*7*).

HBV is transmitted through contact with infected blood or body fluids, such as during pregnancy or delivery, through sex, or by injection drug use (IDU), with the greatest risk for chronic infection occurring during perinatal infection (*8*). Hepatitis B (HepB) vaccination is highly effective in preventing HBV infection and subsequent liver disease; however, 70% of adults in the United States self-reported they were unvaccinated as of 2018 (*9*). Although treatment is not considered curative, antiviral treatment, monitoring, and liver cancer surveillance can reduce morbidity and mortality (*10*,*11*).

To provide a framework for reaching the World Health Organization’s viral hepatitis elimination goals, the *Viral Hepatitis National Strategic Plan for the United States* calls for an increase in the proportion of persons with HBV infection who are aware of their infection from 32% (2013–2016) to 90% by 2030 (*12*,*13*). In support of this goal, this report updates the 2008 CDC recommendations for risk-based testing and management of persons with chronic HBV infection in the United States (*14*). This report is a resource to advise health care professionals, public health officials, and organizations supporting awareness, prevention, and linkage to care about who to screen for HBV infection and which groups at risk for infection to test periodically (Box 1).

BOX 1 **Hepatitis B virus screening and testing recommendations — CDC, 2023**

**Universal hepatitis B virus (HBV) screening**

- HBV screening at least once during a lifetime for adults aged ≥18 years (new recommendation)
- During screening, test for hepatitis B surface antigen (HBsAg), antibody to HBsAg, and total antibody to HBcAg (total anti-HBc) (new recommendation)

**Screening pregnant persons**

- HBV screening for all pregnant persons during each pregnancy, preferably in the first trimester, regardless of vaccination status or history of testing*
- Pregnant persons with a history of appropriately timed triple panel screening and without subsequent risk for exposure to HBV (i.e., no new HBV exposures since triple panel screening) only need HBsAg screening

**Risk-based testing**

- Testing for all persons with a history of increased risk for HBV infection, regardless of age, if they might have been susceptible during the period of increased risk^†^
- Periodic testing for susceptible persons, regardless of age, with ongoing risk for exposures, while risk for exposures persists^†^

* **Source:** Schillie S, Vellozzi C, Reingold A, et al. Prevention of hepatitis B virus infection in the United States: recommendations of the Advisory Committee on Immunization Practices. MMWR Recomm Rep 2018;67(No. RR-1):1–31.

^†^ Susceptible persons include those who have never been infected with HBV (i.e., total anti-HBc negative) and either did not complete a HepB vaccine series per Advisory Committee on Immunization Practices recommendations or who are known to be vaccine nonresponders.

### Interpretation of Screening Tests

**FIGURE 1:**
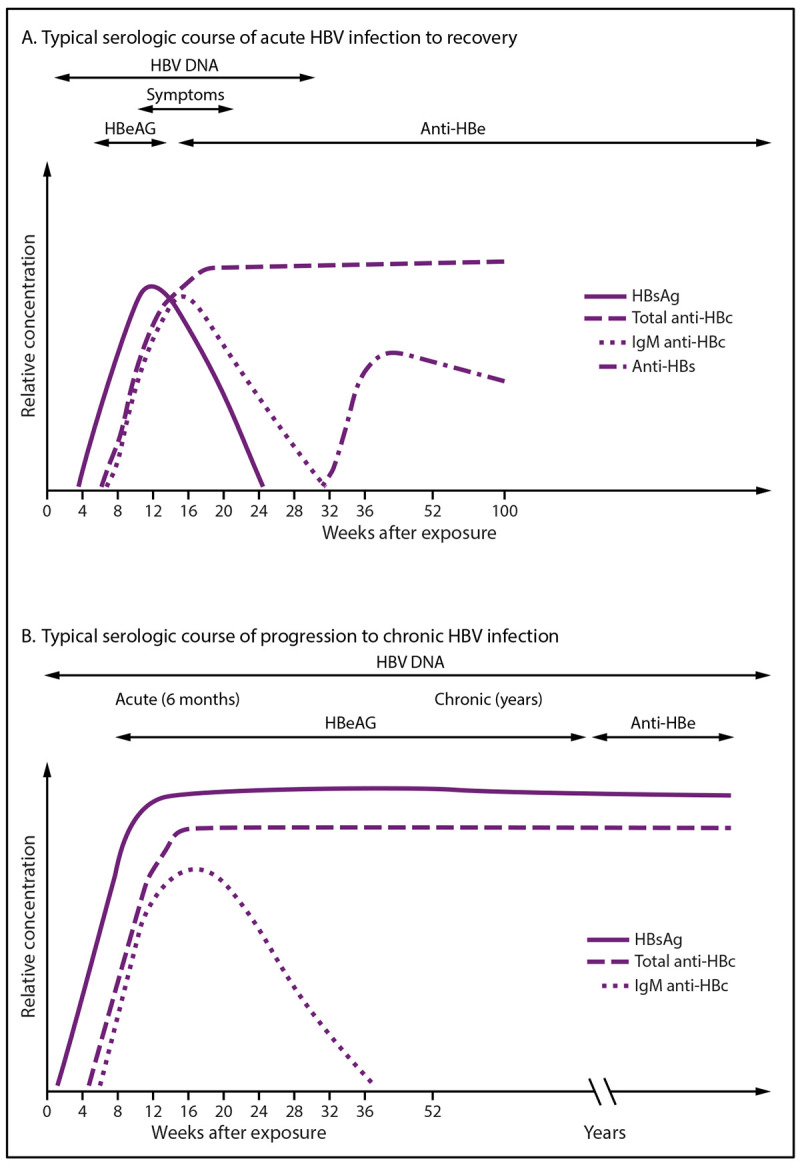
Typical serologic courses of acute and chronic hepatitis B virus infection **Source:** Adapted from Weinbaum CM, Williams I, Mast EE, et al.; CDC. Recommendations for identification and public health management of persons with chronic hepatitis B virus infection. MMWR Recomm Rep 2008;57(No. RR-8):1–20. **Abbreviations:** anti-HBc = antibody to hepatitis B core antigen; anti-HBe = antibody to hepatitis B e antigen; anti-HBs = antibody to hepatitis B surface antigen; HBeAg = hepatitis B e antigen; HBsAg = hepatitis B surface antigen; HBV = hepatitis B virus; IgM = immunoglobulin M.

The three main serologic markers used to determine HBV infection status are hepatitis B surface antigen (HBsAg), antibody to hepatitis B surface antigen (anti-HBs), and antibody to hepatitis B core antigen (anti-HBc) (Table 1). Serologic markers change over typical courses of resolved acute infection and progression to chronic infection (Figure 1) (*15*).

**TABLE 1:** Interpretation of screening test results for hepatitis B virus infection and recommended actions

Clinical state	HBsAg	Anti-HBs	Total anti-HBc*	IgM anti-HBc	Action^†^
Acute infection	Positive	Negative	Positive	Positive	Link to HBV infection care
Chronic infection	Positive	Negative	Positive	Negative^§^	Link to HBV infection care
Resolved infection	Negative	Positive	Positive	Negative	Counsel about HBV infection reactivation risk
Immune (immunity inferred from receipt of previous vaccination)	Negative	Positive^¶^	Negative	Negative	Reassure if history of HepB vaccine series completion; if partially vaccinated, complete vaccine series per ACIP recommendations
Susceptible, never infected	Negative	Negative**	Negative	Negative	Offer HepB vaccine per ACIP recommendations
Isolated core antibody positive^††^	Negative	Negative	Positive	Negative	Depends on cause of positive result

**Abbreviations:** ACIP = Advisory Committee on Immunization Practices; anti-HBs = antibody to hepatitis B surface antigen; HBcAg = hepatitis B core antigen; HBsAg = hepatitis B surface antigen; HBV = hepatitis B virus; HepB = hepatitis B; IgG = immunoglobulin G; IgM anti-HBc = immunoglobulin M antibodies to hepatitis B core antigen; total anti-HBc = total antibody to hepatitis B core antigen.

* Total anti-HBc is a measure of both IgM and IgG antibodies to HBcAg.

^†^ **Source:** Abara WE, Qaseem A, Schillie S, et al. Hepatitis B vaccination, screening, and linkage to care: best practice advice from the American College of Physicians and the Centers for Disease Control and Prevention. Ann Intern Med 2017;167:794–804.

^§^ IgM anti-HBc also might be positive in persons with chronic infection during severe HBV infection flares or reactivation.

^¶^ Immune if anti-HBs concentration is >10 mIU/mL after vaccine series completion.

** Anti-HBs concentrations might wane over time among vaccine responders (**Source:** Schillie S, Vellozzi C, Reingold A, et al. Prevention of hepatitis B virus infection in the United States: recommendations of the Advisory Committee on Immunization Practices. MMWR Recomm Rep 2018;67[No. RR-1]:1–31).

^††^ Can be the result of a past infection when anti-HBs levels have waned, occult infection, passive transfer of anti-HBc to an infant born to an HBsAg-positive gestational parent, a false positive, or mutant HBsAg strain that is not detectable by laboratory assay.

- **HBsAg:** The presence of HBsAg indicates HBV infection, either acute or chronic, except when it might be transiently positive shortly after a dose of HepB vaccine (*16*). The American Association for the Study of Liver Diseases (AASLD) defines chronic infection as the presence of HBsAg for at least 6 months (*11*).
- **Anti-HBs:** The appearance of anti-HBs after a decline of HBsAg indicates recovery from HBV infection. Among immunocompetent persons never infected with HBV, anti-HBs at concentrations of ≥10 mIU/mL at 1–2 months after completion of a HepB vaccine series indicates immunity. Although certain persons might have anti-HBs of ≥10 mIU/mL after partial vaccination, whether this confers long-term protection is unknown. Among vaccine responders who completed a vaccine series, anti-HBs can decline over time to levels of <10 mIU/mL; however, the majority are still immune and will mount an immune response to a vaccine challenge ≥35 years after vaccination (*17*–*20*). Hepatitis B immune globulin (HBIG) can provide anti-HBs for 4–6 months after administration; therefore, testing for anti-HBs ≤6 months after HBIG administration is not an accurate measure of a person’s immune status (*21*).
- **Total anti-HBc:** Total anti-HBc develops in all HBV infections, resolved or current, and typically persists for life. Persons whose immunity to HBV is from a vaccine do not develop anti-HBc. Assays for total anti-HBc detect both immunoglobulin M (IgM) and immunoglobulin G (IgG) antibodies to HBcAg; no test for IgG anti-HBc alone is commercially available. During the typical course of chronic infection, total anti-HBc and HBsAg will be present, whereas IgM anti-HBc will disappear (Figure 1). IgM anti-HBc should be ordered only when acute HBV infection is a concern.
- **Other markers (HBV DNA, HBeAg, and anti-HBe):** HBV DNA is a measure of viral load. HBeAg is a marker for viral replication and high infectivity; antibody to HBeAg (anti-HBe) can be used to monitor response to treatment and chronic HBV infection progression. After identifying a person with HBV infection, testing for HBeAg, anti-HBe, and HBV DNA can provide information on the level of viral replication and infectivity and help guide clinical management.

Background information on HBV, including virus description, transmission, clinical features, natural history, and HepB vaccination seroprotection and coverage, is available (Supplementary Appendix 1, https://stacks.cdc.gov/view/cdc/124432).

### Epidemiology and Risk Factors

#### Acute HBV Infection

Of 3,192 acute HBV infection cases reported to CDC in 2019, an estimated 20,700 new infections (95% CI = 11,800–50,800) were identified after adjusting for underascertainment and underreporting. During 2012–2019, the number of reported acute HBV infection cases in the United States remained relatively stable (*22**,**23*).

Geographic differences exist, with the highest rate of cases (≥2.5 per 100,000 persons) in 2019 reported by Florida, Indiana, Kentucky, Maine, Ohio, Tennessee, and West Virginia (*23*). From 2011 to 2017, the percentage of acute HBV infections among women of childbearing age was stable nationally but increased in Alabama (from 0% to 0.3%), Indiana (from 0% to 0.1%), and Kentucky (from 0.1% to 0.2%) (*24*). Geographic differences in new infections might be because of the opioid crisis; during 2006–2013, increases in incident cases of acute HBV infection in Kentucky, Tennessee, and West Virginia were among persons who reported IDU as a risk factor (*25*).

During 2019, the overall rate of reported acute infections in the United States was 1.0 per 100,000 population. The rate of reported acute HBV infections among persons aged 0–19 years has remained at ≤0.1 case per 100,000 population since 2006, in part because of routine childhood vaccination (*23*). However, transmission of HBV infection persists among adults, especially among older adults for whom vaccine uptake is suboptimal.

Rates of acute HBV infection were higher among males (1.3 per 100,000 population) than females (0.7) and were highest among not Hispanic or Latino (non-Hispanic) White (1.0) persons and non-Hispanic Black persons (0.9). Among the 1,780 case reports that included risk information for IDU, 35% reported IDU (*23*). Among the 1,042 case reports that included sex partner data, 23% reported multiple sex partners. Of the 2,009 case reports that included any risk information, 47% had no risk identified.

#### Chronic HBV Infection

Data from the National Health and Nutrition Examination Survey (NHANES) indicated an estimated 880,000 persons were living with chronic HBV infection during 2013–2018 (95% CI = 580,000–1,170,000) (*5*). The prevalence of resolved HBV infection or HBV infection was 11.7 million persons (95% CI = 10.2–13.5 million). NHANES does not include institutionalized populations and might underestimate the prevalence among ethnic minority groups that are not well represented in the survey. In a 2018 meta-analysis of prevalence, of the estimated 1.89 million persons (range = 1.49–2.40 million) chronically infected with HBV living in the United States, 0.42 million (range = 0.28–0.67 million) were U.S. born and 1.47 million (95% CI = 1.21–1.73) were non-U.S. born (*6*,*26*). By region, the highest proportions of persons with chronic HBV infection in the United States were born in East Asia, Southeast Asia, the Caribbean, South Central Asia, and West Africa (*6*).

From 2011 to 2017, the percentage of chronic HBV infection among women of childbearing age who were tested for HBV infection increased in Kentucky (from 0.2% to 0.4%), Mississippi (from 0.2% to 0.4%), and West Virginia (from 0.3% to 0.4%) (*24*). In 2019, the rate of newly reported cases of chronic HBV infection among adults varied by age, with the highest rate (11.3 per 100,000 persons) reported among persons aged 30–39 years and the lowest rate (0.5) reported among persons aged 0–19 years (*23*). During 2015–2017, an estimated 20,678–21,314 infants were born to pregnant women who were HBsAg positive (*27*). National Perinatal Hepatitis B Prevention Program data indicated that only half (52.6%) of these infants were identified through prenatal screening in 2017.

During 2019, a total of 1,662 deaths attributable to HBV infection in the United States were reported on death certificates, resulting in an age-adjusted rate of 0.42 per 100,000 persons (95% CI = 0.40–0.44) (*23*). The highest death rates occurred among Asian and other Pacific Islander persons (2.10), males (0.66), and persons aged 65–74 years (1.54). However, deaths attributable to HBV infection have been found to be underreported on death certificates (*1*).

## Methods

This report updates and expands CDC recommendations for hepatitis B screening of adults published in 2008 (*14*). CDC evaluated the addition of a universal screening recommendation among adults as well as testing persons expected to be at increased risk for HBV infection that were not included in the 2008 testing recommendations.

Members of the CDC Guidelines Work Group (hereafter referred to as the work group) followed CDC guideline development and reporting standards (*28*) to develop research questions needed to assess the proposed updates; conduct systematic reviews; assess the quality of the evidence; and review existing systematic reviews, meta-analyses, and cost-effectiveness analyses, when available (Supplementary Appendix 2; Supplementary Tables 1, 4, and 7, https://stacks.cdc.gov/view/cdc/124432). Comprehensive systematic literature reviews were conducted for recommendations on 1) expanding screening to all adults (i.e., universal screening), 2) periodic testing for HBV infection among persons with hepatitis C virus (HCV) infection, and 3) testing for HBV infection among persons with a history of incarceration.

For all three systematic reviews, literature searches were conducted by CDC librarians with direction from subject matter experts. Searches were conducted for English-language literature published worldwide in Medline (OVID), Embase (OVID), CINAHL (Ebsco), and Cochrane Library. Duplicates were identified and removed using Endnote (version 20; Clarivate Analytics) and DistillerSR systematic review software (version 2.35; Evidence Partners) automated “find duplicates” functions.

CDC’s Viral Hepatitis Steering Committee considered multiple methods to assess quality of evidence. The Mixed Methods Appraisal Tool (MMAT) was selected because it is a validated tool for assessing nonrandomized analytic and descriptive studies, which comprise most of the HBV infection prevalence literature (*29*). MMAT users rate each study on methodological quality criteria, indicating whether criteria were met with “Yes,” “No,” or “Can’t Tell.” Calculating a summary score is not recommended for the tool because presenting a single number is not informative about which aspects of the studies are problematic. Economic analyses were evaluated by assessing whether the study met the Consolidated Health Economic Evaluation Reporting Standards (CHEERS) (*30*). 

CDC determined that the new recommendations constituted influential scientific information that will have a clear and substantial impact on important public policies and private sector decisions. Therefore, the Information Quality Act required peer review by specialists in the field who were not involved in the development of these recommendations. CDC solicited nominations for reviewers from AASLD, the Infectious Disease Society of America, and the American College of Physicians (ACP). Five clinicians with expertise in hepatology, gastroenterology, internal medicine, or infectious diseases provided structured peer reviews and any edits made in response were documented (Supplementary Appendices 2 and 3, https://stacks.cdc.gov/view/cdc/124432). No CDC staff or external peer reviewers reported a conflict of interest. In addition, feedback from the public was solicited through a *Federal Register* notice announcing the availability of the draft recommendations for public comment from April 4 through June 3, 2022. CDC received 28 public comments on the draft document from nonprofit/advocacy groups, providers, industry groups, medical professional organizations, the public, academia, and a consulting group. Public comments were considered by the work group and any edits made in response were documented (Supplementary Appendix 4, https://stacks.cdc.gov/view/cdc/124432). 

The work group also presented these guidelines to the CDC/Health Resources and Services Administration (HRSA) Advisory Committee on HIV, Viral Hepatitis and STD Prevention and Treatment, but did not seek consensus decision-making from this advisory committee. The steering committee considered results of the systematic reviews in conjunction with cost-effectiveness analyses, supplemental literature, practicality of implementing guidelines, public health benefits, subject matter expertise, and reviewer and public feedback.

### Systematic Review Methods

#### Universal Screening

The search period was January 1, 2008 (the year of the last CDC screening guidelines) through February 8, 2021 (Supplementary Table 2, https://stacks.cdc.gov/view/cdc/124432). Search results were supplemented by relevant studies identified through reference lists in review articles and by newly published studies. DistillerSR was used to organize the review process. Each article was reviewed for inclusion by two of the authors (EC and LP). Differences in decisions to include were discussed until consensus was reached. Articles were included if they contained the prevalence or incidence of HBV infection among adults aged ≥18 years or linkage-to-care data. Articles were excluded if they were conducted outside the United States and U.S. territories; only reported data from a study not conducted in humans, environmental studies, or technology assessments; lacked original data (e.g., editorials, reviews, and modeled data); were case reports; or only included self-reported (i.e., unconfirmed) HBV infection prevalence (Supplementary Table 3, https://stacks.cdc.gov/view/cdc/124432). When a reviewer identified an article as meeting any exclusion criterion, additional exclusion criteria were not assessed or recorded. When multiple articles reported data on the same cohort, only the article with the most complete data was included. Data were independently abstracted by two reviewers (EC and LP) and discrepancies were discussed until consensus was reached or they were resolved by a third reviewer (NN). Finally, two independent assessors (LP, JB, or NN) used MMAT to assess the quality of articles used to calculate the prevalence of HBV infection in the general population.

#### Persons with HCV Infection

The search period was January 1, 2005 through September 22, 2020 (Supplementary Table 5, https://stacks.cdc.gov/view/cdc/124432). DistillerSR and Endnote were used to organize the review process. Titles were reviewed by one reviewer (PS or EC), and those that were clearly irrelevant to the research question were excluded. Each potentially relevant article was reviewed for inclusion by two of the authors (MH and PS). Differences in decisions to include were discussed until consensus was reached (Supplementary Table 6, https://stacks.cdc.gov/view/cdc/124432). Data from the included full text articles were independently abstracted by two reviewers (MH, PS, or EC). The quality of the articles was assessed using MMAT. The population was considered at “increased risk” if the prevalence of HBV infection was ≥1%.

#### Persons with a History of Incarceration in a Jail, Prison, or Other Detention Setting

The work group used an existing literature search of articles on HBV and HCV infections in correctional and detention facilities. The search period was January 1, 2000 through March 3, 2021 (Supplementary Table 8, https://stacks.cdc.gov/view/cdc/124432). Abstracts were reviewed by two reviewers (AH, LH, JB, OR, or EC) for relevance, and discrepancies in inclusion were resolved by the first author (EC) or by consensus discussion. Only articles containing incidence or prevalence of HBV infection among persons with a history of incarceration or incarceration as a risk factor for HBV infection were included in this review (Supplementary Table 9, https://stacks.cdc.gov/view/cdc/124432). Data from the included full text articles or abstracts were independently abstracted by two reviewers (LP and EC) and differences were resolved by consensus discussion. Because of the limited amount of literature available about HBV infection in correctional settings, the work group included conference abstracts, which are labeled as such because of their presumed lower quality. The quality of the articles was assessed using MMAT. The population was considered at “increased risk” if the prevalence of HBV infection was ≥1%.

## Universal Screening Systematic Review and Review of Evidence Summary

After deduplication, 2,580 records were available for initial title screen; 1,374 articles were excluded during title screen. An additional 1,028 articles were excluded during abstract review. Among the 178 full text articles, 136 did not meet inclusion criteria after review; 42 articles were included in the final review (Supplementary Table 11, https://stacks.cdc.gov/view/cdc/124432). An additional article met inclusion criteria, but was published after the search period, and was abstracted to supplement evidence from the systematic search.

Eighteen articles had any HBV testing data from the general population (i.e., screening persons not known to be at increased risk for HBV infection) (Supplementary Table 11, https://stacks.cdc.gov/view/cdc/124432). Testing recommendations are risk based; therefore, studies with convenience samples of persons already tested for HBV infection were considered biased toward overestimating the prevalence of HBV infection even if the study did not explicitly state that there was risk-based testing. The remainder of the articles (n = 25) included persons at increased risk for HBV infection who were not considered to be representative of the general U.S. population. The individual MMAT quality ratings are available (Supplementary Tables 14 and 15, https://stacks.cdc.gov/view/cdc/124432).

### Key Research Questions

Q1: How would adult universal screening for hepatitis B affect the number (and composition) of persons who screen positive for HBV infection?
Q1a: What is the prevalence of chronic HBV infection in the United States? In the general population, by age groups?

The work group defined patients who have chronic HBV infection as those who were HBsAg positive, except for one study in which authors classified patients as having chronic HBV infection without providing a definition. Studies among first-time blood donors, organ donors, pregnant women (among whom universal screening is already recommended), NHANES enrollees, and patients seeking care for a condition other than HBV infection were included. 

On the basis of 17 studies conducted both in the United States and U.S. territories, the median prevalence of chronic HBV infection in the general population was 0.4% (range = 0.0%–2.0%) (Supplementary Table 11, https://stacks.cdc.gov/view/cdc/124432). On the basis of studies conducted in the United States alone, the prevalence was 0.38% (range = 0%–0.74%) (Supplementary Table 11, https://stacks.cdc.gov/view/cdc/124432). Eight studies reported the prevalence of a history of infection (i.e., anti-HBc positive, HBsAg negative); the median was 6.2% (range = 4.8%–14.0%) (*31*–*38*).

The ages of patients with chronic HBV infection (when available) are included in the summary table (Supplementary Table 11, https://stacks.cdc.gov/view/cdc/124432). No clear trends were identified in the prevalence of chronic HBV infection by age across studies. Therefore, the work group considered the economic analysis, vaccination rates and efficacy, the epidemiology of acute and chronic infections from surveillance data, ease of implementation, and harms of missed identification of chronic infections in determining the age thresholds for universal adult screening.

Q1b: What is the yield (number of new diagnoses per tests completed) and sensitivity of alternative HBV screening strategies (e.g., universal versus targeted screening or screening strategies based on alternative risk factors)?

As part of their HBV screening recommendations systematic review, the U.S. Preventive Services Task Force (USPSTF) assessed the yield (number of new diagnoses per tests completed) and sensitivity of alternative HBV infection screening strategies (*39*). USPSTF identified three fair quality, non-U.S.–based studies, which might limit applicability (*40*–*42*). On the basis of these studies, the number of persons who need to be screened to identify one HBV infection using risk-based strategies ranged from 32 to 148. In comparison, fewer than 20 persons need to be screened to identify a case of HCV infection using risk-based screening (*43*).

Only one of the studies, conducted in France, assessed CDC’s risk-based testing criteria (*41*). Using risk-based testing had 100% sensitivity (i.e., 100% of infected persons were identified), and self-report identified 70% of persons with at least one risk factor; however, the study population specifically overrepresented persons at increased risk for infection.

The work group also considered a prospective cohort study of patients with cancer at one U.S. health center, where applying CDC risk criteria to screening had 97% sensitivity (*44*). The proportion of patients who met at least one risk criterion was 91%. Therefore, in terms of provider time, universal screening might be more efficient than risk-based testing. Because no studies directly assessed universal screening, the work group could not provide the yield of universal screening versus risk-based screening.

Q2: How many additional persons would be linked to care?
Q2a: What is the diagnostic accuracy of HBV testing?

The diagnostic accuracy of HBV tests has been evaluated by the Food and Drug Administration (FDA) and was not included as part of the systematic review. Any assay that receives FDA approval for clinical use must meet high standards of diagnostic accuracy. A list of FDA-approved HBV serologic assays, including links to detailed information on their performance characteristics, is available (Supplementary Table 21, https://stacks.cdc.gov/view/cdc/124432).

Q2b: What are the harms of hepatitis B screening?

Data on harms in the systematic review were limited. In one study, women with public insurance and who self-paid for health care services were less likely to be screened, even though HBsAg screening costs should have been covered; the authors hypothesized that out-of-pocket payments might be a barrier to screening (*45*). In another study assessing acceptability of hepatitis screening among patients during colonoscopies, acceptance was 78% (*46*).

Harms of screening for HBV would be expected to be similar to those for HCV. In a previous review, possible harms of screening for hepatitis C were physical pain, anxiety, cost, interpersonal problems related to learning infection status, stigma, time, fear, and reluctance to disclose illegal risk behaviors (*47*). Other plausible harms included concern caused by false-positive results, distress resulting from lack of education or understanding of resolved infection, insurability and employment issues, and treatment adverse effects.

The work group concluded that potential harms of screening did not outweigh the benefits. In addition, universal screening might reduce harms compared with risk-based screening by not requiring persons to disclose potentially stigmatizing risk conditions (e.g., immigration status and IDU) to get tested.

Q2c: What proportion of persons who screen positive for HBV infection are linked to care?
Q2d: What proportion of persons who screen positive for HBV infection are treatment eligible?
Q2e: What proportion of eligible persons who screen positive for HBV infection are treated?

Only two studies from the universal screening review reported on linkage to care. In a study among persons attending free clinics, 69% of patients with a diagnosis of chronic HBV infection enrolled in follow-up care (*48*). In a free screening clinic, 78% of patients with HBV infection elected to undergo follow-up monitoring (i.e., alanine aminotransferase [ALT]) and HBV DNA), and 24% (11 of 45) of those monitored were eligible for treatment (i.e., viral load of >20,000 copies per mL) (*49*).

Data on treatment were only available in two studies of antiviral treatment during chemotherapy. In one study, 23% of patients at risk for reactivation were prescribed a preventive nucleoside analog (*50*). In the other study, 12% (18 of 152) of patients with a previous HBV infection received antiviral drugs, and 73% (11 of 15) of patients with chronic HBV infection received antiviral drugs (*36*).

To answer these key questions, the work group also assessed evidence from two additional studies that were not part of the systematic review but included the general population. In a 2008–2016 study of adults with chronic HBV infection and commercial insurance, 36% (6,004 of 16,644) of patients were linked to care (defined as having had an ALT test and HBV DNA or HBeAg test) (*51*). Of the patients with chronic HBV infection with prescription claims, 18% (2,926 of 16,572) were treated. Among 2,338 patients with chronic HBV infection followed in a prospective cohort study, 78% had one or more ALT tests annually, 37% had one or more HBV DNA tests annually, and 32% were treated (*52*). Not all patients with chronic HBV infection require treatment; estimates of patients with HBV infection meeting AASLD criteria for treatment range from 24% to 48% (*53**,**54*). These two studies did not assess the proportion of persons treated among those who were eligible. Overall, the work group found that linkage-to-care rates ranged from 36% to 78%, and from 18% to 32% of patients with chronic HBV infection were prescribed treatment.

Q3: How many new infections of HBV would be prevented?
Q3a: What proportion of close contacts are at risk for infection?

The work group did not identify evidence directly assessing the proportion of close contacts (excluding perinatal transmission) who are at risk for infection and thus could not estimate the proportion of new infections that would be prevented by universal adult screening. However, the work group found evidence of the proportion of close contacts of persons with HBV infection who themselves have HBV infection.

From the systematic review, a cohort study of patients with cancer and previous HBV infection found that 8.1% reported having a household contact with HBV infection (who was not a sex partner), and 15.2% reported sexual contact with a person with HBV infection. Of the patients with chronic HBV infection, 0.5% reported a nonsexual household contact with HBV infection, and 1.5% reported sexual contact with a person with HBV infection (*36*).

In a study of programs testing and linking patients with hepatitis B to care in the United States, 14% of household contacts of persons who were HBsAg positive were themselves HBsAg positive, and 30% had a history of infection (anti-HBc positive) (*55*). In 2019, surveillance data indicated that 10% (92 of 899) of persons with acute cases had a sexual contact and 2% (17 of 899) had a nonsexual household contact (*23*). However, relying on self-reports of close contacts with HBV infection likely underestimates the risk. Global studies conducted during 1974–2007 found that 14%–60% of persons living in households with persons with chronic HBV infection have serologic evidence of resolved HBV infection, and 3%–30% have chronic infection (*14*). Although screening can prevent further spread of HBV infection, the work group was unable to estimate the size of that impact.

Q4: Do desirable management and treatment effects outweigh undesirable effects?

Key Q4 was not assessed by the systematic review because it has been reported elsewhere. USPSTF reviewed effectiveness of treatment on reducing viral load, HBeAg, HBsAg, cirrhosis, hepatocellular carcinoma (HCC), and death (*39*). Antiviral therapy was associated with viral suppression, HBsAg loss, normalization of ALT levels, and HBeAg loss. Antiviral therapy was associated with decreased risk for HCC and death compared with placebo or no therapy; however, data were sparse and estimates imprecise. Therapy was not associated with an increased risk for serious adverse events. The conclusion of AASLD’s systematic review used in the development of its treatment guidelines was that recommended treatment reduces cirrhosis, decompensated cirrhosis, HCC, and death in adults with active chronic HBV infection and is strongly recommended (*10*).

## Cost-Effectiveness of Screening Strategies

### Universal Screening

A 2021 economic analysis on the cost-effectiveness of one-time universal HBV screening of adults aged 18–69 years provided information for these guidelines (*56*). With an estimated prevalence of undiagnosed chronic HBV infection of 0.24%, universal HBsAg screening among adults aged 18–69 years was cost-saving compared with current practice, assuming antiviral treatment drug costs remain at <$894 per year. Antiviral treatment drug costs would need to rise to $9,692 a year (approximately 19 times the cost at the time of the study) for universal screening to be no longer cost-effective. Undiagnosed prevalence was based on the NHANES estimate of 0.36% and the finding that 67% of persons with HBV infection were unaware of their infection (*57*). Current practice was based on the literature and assumed that 33% of people with HBV infection were currently diagnosed, 36% were linked to care, and 18% were receiving treatment (*56*). 

Compared with current practice, universal screening would be expected to avert an additional 7.4 cases of compensated cirrhosis, 3.3 cases of decompensated cirrhosis, 5.5 cases of HCC, 1.9 liver transplants, and 10.3 HBV-related deaths per 100,000 persons screened (*56*). Universal HBsAg screening of adults aged 18–69 years would save $262,857 per quality-adjusted life year (QALY) and would result in a gain of 135 QALYs per 100,000 adults screened. A probabilistic sensitivity analysis that varied all parameters in the model simultaneously indicated a >99% likelihood that universal screening would be cost-effective compared with current practice at a maximum willingness-to-pay threshold of $50,000 per QALY.

Study authors conducted an unpublished analysis using the same methods as those in the economic analysis described in this report, but with an upper age limit of 80 years instead of 69. They found one-time universal screening of adults aged 18–80 years with an HBsAg test would save $200,334 and result in a gain of 128 QALYs per 100,000 adults screened.

A sensitivity analysis found that using the triple panel (HBsAg, anti-HBc, anti-HBs) and assuming Medicare reimbursement of $28.27, universal screening with the triple panel would be cost-effective, with an incremental cost-effectiveness ratio of $11,207 per QALY (*56*). Using a cost-effectiveness threshold of $50,000 per QALY, universal screening with the triple panel remained cost-effective if the HBV infection prevalence was >0.15%. A summary of the CHEERS checklist is available (Supplementary Table 20, https://stacks.cdc.gov/view/cdc/124432). Minor deviations from the recommended standards were not considered a substantial risk to quality. 

### Screening in Higher Prevalence Settings

A 2022 cost-effectiveness analysis evaluated whether screening in STI clinics (i.e., a high-prevalence setting) with universal vaccination can reduce costs and improve care (*58*). The researchers assumed the study population was aged 18–69 years, had an estimated HBsAg prevalence of 4.2%, and had no previous HepB vaccination or known HBV infection. One-time screening with the triple panel was cost-saving and prevented an additional 138 cases of cirrhosis, 47 cases of decompensated cirrhosis, 90 cases of HCC, 33 liver transplants, and 163 HBV-related deaths per 100,000 adults screened. Even if chronic HBV infection prevalence in the STI clinic population was assumed to be zero, screening plus vaccination was less costly than vaccination alone because it identified persons with previous vaccination and averted the cost of additional vaccine doses.

## Universal Screening Summary of Findings

The steering committee considered results of the systematic review in conjunction with cost-effectiveness analyses, supplemental literature, practicality of implementing guidelines, public health benefits, subject matter expertise, and reviewer and public feedback. Because of limited data, the steering committee was only indirectly able to assess the key question “How would adult universal screening for hepatitis B affect the number [and composition] of persons who screen positive for HBV infection?” A summary of the evidence considered, rationale for screening (Box 2), conclusions of the steering committee, and limitations is available (Supplementary Table 10, https://stacks.cdc.gov/view/cdc/124432). The steering committee concluded that simplifying the implementation of screening from a risk-based to a universal approach might increase the number of persons aware of their infection. Overall, risk-based testing has been insufficient to identify persons with HBV infection in the United States and has been a barrier to appropriately screening populations with a disproportionate prevalence of disease. Assessment of risk is difficult for providers and might be stigmatizing to the patient.

BOX 2 **Rationale for universal hepatitis B virus screening**

- Hepatitis B virus (HBV) infection has substantial morbidity and mortality.
- Chronic HBV infection can be detected before the development of severe liver disease using reliable and inexpensive screening tests.
- Treatment for chronic HBV infection can reduce morbidity and mortality.
- Management of chronic HBV infection might prevent transmission to others.
- Universal screening of adults is cost-effective.
- Screening enables identification and management of pregnant persons infected with HBV and their infants, which can reduce the risk for perinatal transmission.
- Screening can identify persons who are at risk for reactivation of HBV infection.
- Screening might identify persons who would benefit from hepatitis B vaccination.

A one-time HBV screening of adults would be complementary to the 2022 Advisory Committee on Immunization Practices (ACIP) recommendation to vaccinate all adults aged 19–59 years for HBV infection because screening establishes any history of infection, and vaccination protects from future infection and need for additional testing (*59*). The recommendations were supported by peer reviewers who are experts in the field as well as the majority of public comments. Patients with HBV infection have increased morbidity and mortality, and monitoring and treatment can improve health outcomes. If more efficacious treatments are approved in the future, this benefit will increase further. Although increasing awareness of infection is expected to reduce transmission to close contacts, this assumption is hypothetical because of the lack of direct evidence. No studies directly compared universal screening with risk-based screening; therefore, the steering committee relied on the cost-effectiveness study finding that a one-time universal screen of adults is cost-effective and results in improved health outcomes as compared with risk-based screening (*56*).

### Persons with an Increased Risk for HBV Infection Recommended for Testing

#### Persons with HCV Infection or a Past HCV Infection 

The systematic review found 8,295 articles for review; after title review, 1,233 potentially relevant articles remained. After review of articles meeting inclusion and exclusion criteria, 17 articles were included (Supplementary Table 12, https://stacks.cdc.gov/view/cdc/124432). In 10 U.S. studies, the prevalence of current HBV infection (on the basis of HBsAg positivity, HBV DNA positivity, or *International Classification of Diseases, Tenth Revision* codes) among persons with HCV infection ranged from 0.2% to 5.8% (median = 1.2%) (*60*–*69*). Among persons with HCV infection, the prevalence of ever being exposed to HBV ranged from 24.7% to 62.6% (median = 43.0%); this finding was based on anti-HBc positivity, regardless of other HBV test results (*62*–*65*,*69*,*70*). Isolated anti-HBc positivity ranged from 36.9% to 53.8% (median = 39.5%) among patients with HCV infection (*62*,*65*,*69*).

##### HBV Reactivation During Direct-Acting Antiviral Therapy for HCV

FDA requires a boxed warning about the risk for HBV reactivation to be added to drug labels of direct-acting antiviral (DAA) medication for HCV infection. The boxed warning directs health care professionals to screen and monitor for HBV infection in all patients receiving DAA treatment (*71*).

In a published systematic review of HBV reactivation during DAA therapy among patients with HCV infection, the overall risk for HBV reactivation was 24% (95% CI = 19%–30%) in patients with untreated chronic HBV infection and 1.4% (95% CI = 0.8%–2.4%) in patients with resolved HBV infection (*72*). The risk for HBV reactivation–related hepatitis (i.e., symptomatic) was 9% (95% CI = 5%–16%) in patients with chronic HBV infection; HBV reactivation–related hepatitis did not occur in patients with resolved infection. Three of 1,621 patients with chronic HBV infection had a major clinical event related to the reactivation (liver decompensation or failure), but there were no deaths.

Four studies (*62**,**69**,**73**,**74*) were published after the 2018 systematic review (*72*). In two national cohort studies of U.S. veterans with chronic HCV infection prescribed DAA therapy, HBV reactivation was rare (<0.1%) and more frequent among patients who were HBsAg positive (*62*,*73*). Similarly, two other U.S.-based cohort studies of patients with HCV coinfected with HBV did not detect any cases of DAA-associated HBV reactivation (*69*,*74*).

##### Outcomes of HCV/HBV Coinfection

In a study comparing patients with HCV infection achieving sustained virologic response to HCV treatment, anti-HBc positivity was identified as an independent risk factor for the development of HCC (hazard ratio [HR] = 5.57; 95% CI = 1.45–21.39) (*75*). Conversely, a nested, case-control study of patients who were HBsAg negative with HCV infection indicated that neither previous nor occult HBV infection was associated with the development of HCC (*76*). Clinically significant hepatic events, including HBV reactivation, were more common among patients who were cirrhotic than patients who were noncirrhotic anti-HBc positive with chronic HCV infection undergoing DAA therapy (*73*). Among a cohort of 51,781 veterans who were HCV infected and who initiated DAA treatment, those who were HBV/HCV coinfected (odds ratio [OR] = 2.25; 95% CI = 1.17–4.31) and those with resolved HBV infection (OR = 1.09; 95% CI = 1.03–1.15) were more likely to achieve sustained virologic response compared with patients who were HCV monoinfected (*64*).

In a national cohort of 99,548 U.S. veterans, patients with HCV infection and documented HBV viremia (HBV DNA detected) were at significantly higher risk for cirrhosis (adjusted hazard ratio [aHR] = 1.89; 95% CI = 1.46–2.45), HCC (aHR = 2.12; 95% CI = 1.26–3.60), and death (aHR = 1.62; 95% CI = 1.33–1.99) than patients who were HCV monoinfected, after controlling for demographic, clinical, and antiviral treatment–related factors (*68*). In this cohort, absence of HBV replication was associated with a clinical course similar to that of patients who were HCV monoinfected. Compared with patients who were HCV monoinfected, patients with HBV/HCV coinfection had more advanced fibrosis, a faster fibrosis progression rate, and more severe steatosis (*63*). In a matched case-control study, patients with HBsAg-negative HCV infection with HCC were more likely to have had previous HBV infection (anti-HBc positive), regardless of anti-HBs status (anti-HBs negative [OR = 2.98; 95% CI = 2.12–5.08]; anti-HBs positive [OR = 1.84; 95% CI = 1.22–3.08]), compared with HCV-infected controls without HCC (*77*).

Many studies had incomplete test data and used descriptive tests of significance rather than models that controlled for other variables. The results from the MMAT quality assessment are available (Supplementary Tables 18 and 19, https://stacks.cdc.gov/view/cdc/124432). The work group concluded that because the prevalence estimate was ≥1% for HBV infection and because of the boxed warning for DAAs, persons with HCV infection or a past HCV infection should be considered at increased risk for HBV infection.

#### Persons Incarcerated or Formerly Incarcerated in a Jail, Prison, or Other Detention Setting

 The systematic review of HBV infection in correctional settings used for these testing guidelines was part of a larger review that also contained articles on HCV infection in correctional settings (“review 1”). The initial search of literature on HBV infection and HCV infection in correctional settings yielded 2,395 unique articles for review; of these, 1,961 were deemed irrelevant by title and abstract screening, resulting in 434 potential articles for review 1. A secondary abstract review (“review 2”), which applied the inclusion and exclusion criteria for these guidelines, resulted in 57 articles that met the inclusion criteria for full text review; three of these articles also were included in the HBV universal screening systematic review. After full text review, 10 articles were included (Supplementary Table 13, https://stacks.cdc.gov/view/cdc/124432). The individual MMAT quality ratings are available (Supplementary Tables 16 and 17, https://stacks.cdc.gov/view/cdc/124432).

Among eight studies, the prevalence of chronic HBV infection in persons with a history of incarceration ranged from 0.6% to 8.7% (median = 1.0%) (*78*–*85*). Two studies of men who were incarcerated assessed incidence, which ranged from 2,700 to 3,579 infections per 100,000 persons per year (*78*,*81*). One study reported 41 acute HBV infections acquired in prison; however, the total number tested was not reported and therefore a prevalence or incidence rate could not be calculated (*85*). Another study reported an infection rate of 1.2% during an outbreak of HBV infection in a high-security correctional facility (*83*).

Three studies found an increased risk for HBV infection associated with incarceration. In a study of blood donors, persons detained ≥3 nights in a jail or detention facility had three times higher odds of having serologic evidence of HBV infection; however, the comparison group was not provided (p≤0.001) (*86*). In another study, persons incarcerated >14 years had 1.68 (95% CI = 1.08–2.59) higher odds of ever acquiring HBV infection compared with those incarcerated ≤7 years (*81*). Finally, a third study indicated that persons with any self-reported history of incarceration had increased odds (OR = 1.84; 95% CI = 1.02–3.31) of ever having HBV infection compared with persons with no history of incarceration (*87*).

The work group determined that persons incarcerated or formerly incarcerated in a jail, prison, or other detention setting should be considered at increased risk. This conclusion was based on the HBV infection prevalence estimate of ≥1% and the studies directly indicating an association between HBV infection and incarceration. The reasons for increased risk for HBV infection among persons who have been incarcerated might include behaviors that occur before or during incarceration, including drug use, higher-risk sex, percutaneous exposures (e.g., tattooing), and structural factors that affect the level of risk for these behaviors (e.g., availability of condoms, clean syringes, and engagement in health care). To ensure all incarcerated persons receive recommended HBV testing, correctional and detention facilities should consider offering HBV screening at intake, periodic testing for susceptible persons serving long-term sentences, and HepB vaccination for susceptible persons (*16*).

#### Persons with Sexually Transmitted Infections or a History of Sexually Transmitted Infections or Multiple Sex Partners 

The work group used a published systematic review and meta-analysis to assess risk among persons with a history of a non-HIV sexually transmitted infection (STI) (*88*). This analysis of studies worldwide found positive and statistically significant associations between the prevalence of HBV infection and other STIs. Three U.S. studies, published during 2008–2009, included four estimates of HBsAg prevalence among persons with syphilis or any STI; the median prevalence was 1.6% (range = 0.9%–33.2%). Among the four estimates, two were among groups with other risk factors for HBV infection (e.g., persons being processed into jail and men who have sex with men [MSM]). Seven U.S. studies, published during 1998–2000, included nine estimates of prevalence of HBV infection or a history of HBV infection (HBsAg or anti-HBc positive) among persons with STIs or a history of STIs; the median prevalence was 22.4% (range = 8.6%–83.5%). Among the nine estimates of past infection, four were among groups with other risk factors for HBV infection (e.g., persons who use drugs, persons with HIV infection, and MSM).

A study of national surveillance reports and survey data during 2013–2018 found 1,324 (38.2%) cases of sexually transmitted acute HBV infection after excluding cases with a report of IDU; 5.3% of persons reported sexual contact with a person with HBV infection, 3.1% reported being male and having sex with another male partner, 27.8% reported having multiple sex partners, and 2% reported a history of STI treatment 6 weeks to 6 months before their HBV infection diagnosis (*89*). Cases were classified into mutually exclusive categories in the order listed. The work group considered the HBsAg prevalence of >1% among persons with an STI to be sufficient evidence of increased risk. Although the recommendation for multiple partners is not directly supported by the literature, it aligns with AASLD recommendations to screen persons who are not in a long-term, mutually monogamous relationship (i.e., more than one sex partner during the previous 6 months) (*11*).

#### Infants Born to Pregnant Persons Who Are HBsAg Positive

Without preventive steps, 90% of infants born to women who are HBsAg and HBeAg positive and 5%–20% of infants born to women who are HBsAg positive, HBeAg negative will become infected (*90*–*92*). Additional information is available in *Prevention of Hepatitis B Virus Infection in the United States: Recommendations of the Advisory Committee on Immunization Practices* (*15*) and from AASLD (*11*).

#### Persons Born in Regions with HBV Infection Prevalence of ≥2%

A 2021 systematic review and meta-analysis estimated the prevalence of non-U.S.–born persons with chronic HBV infection in the United States to be 3.1% (95% CI = 2.5%–3.6%). Africa had the highest regional prevalence (8.6%), followed by Asia (5.9%) and Oceania (4.5%) (*6*) (Box 3).

BOX 3 **Prevalence of chronic hepatitis B virus infection, by country or territory**

**High prevalence (≥8%):** Angola, Cabo Verde, Central African Republic, Chad, Eswatini, Ghana, Guinea, Guinea-Bissau, Kiribati, Lesotho, Liberia, Mali, Mauritania, Niger, Nigeria, Philippines, Sao Tome and Principe, Sierra Leone, Solomon Islands, Taiwan, Timor-Leste, Togo, Tonga, Turkmenistan, Tuvalu, and Zimbabwe.

**Intermediate prevalence (5%–7.9%):** Albania, Benin, Burkina Faso, Cameroon, China, Côte d’Ivoire, Democratic People’s Republic of Korea, Djibouti, Eritrea, Ethiopia, Federated States of Micronesia, Gabon, Indonesia, Kyrgyzstan, Moldova, Mongolia, Mozambique, Myanmar, Papua New Guinea, Senegal, Somalia, South Sudan, Syria, Tajikistan, Uzbekistan, Vanuatu, and Vietnam.

**Low–intermediate prevalence (2%–4.9%):** Afghanistan, Azerbaijan, Bangladesh, Belarus, Bosnia and Herzegovina, Bulgaria, Burundi, Cambodia, Comoros, Congo, Democratic Republic of Congo, Gambia, Georgia, Guyana, Haiti, Hong Kong, India, Iraq, Jamaica, Jordan, Kazakhstan, South Korea, Laos, Madagascar, Malawi, Malaysia, Marshall Islands, Oman, Pakistan, Romania, Rwanda, Samoa, Singapore, South Africa, Sri Lanka, Sudan, Tanzania, Thailand, Trinidad and Tobago, Tunisia, Turkey, Uganda, Yemen, and Zambia.

**Low prevalence (≤1.9%):** Algeria, Argentina, Armenia, Australia, Austria, Bahrain, Belgium, Belize, Bhutan, Bolivia, Brazil, Canada, Chile, Colombia, Costa Rica, Croatia, Cuba, Czechia, Denmark, Dominican Republic, Ecuador, Egypt, El Salvador, Estonia, Fiji, Finland, France, Germany, Greece, Guatemala, Honduras, Hungary, Iran, Ireland, Israel, Italy, Japan, Kenya, Kosovo, Kuwait, Lebanon, Libya, Mexico, Morocco, Nepal, Netherlands, New Zealand, Nicaragua, Norway, Palestine, Panama, Paraguay, Peru, Poland, Portugal, Qatar, Russia, Saudi Arabia, Slovakia, Slovenia, Spain, Suriname, Sweden, Switzerland, Ukraine, United Arab Emirates, United Kingdom, United States, and Venezuela.

**Unknown prevalence (data not available):** American Samoa, Andorra, Anguilla, Antigua and Barbuda, Aruba, Bahamas, Barbados, Bermuda, Bonaire Sint Eustatius and Saba, Botswana, British Virgin Islands, Brunei, Cayman Islands, Cook Islands, Curaçao, Cyprus, Dominica, Equatorial Guinea, Falkland Islands, Faroe Islands, French Guiana, French Polynesia, Gibraltar, Greenland, Grenada, Guadeloupe, Guam, Holy See, Iceland, Isle of Man, Latvia, Liechtenstein, Lithuania, Luxembourg, Macao, Macedonia, Maldives, Malta, Martinique, Mauritius, Mayotte, Monaco, Montenegro, Montserrat, Namibia, Nauru, New Caledonia, Niue, Northern Mariana Islands, Palau, Puerto Rico, Réunion, Saint Barthélemy, Saint Helena, Saint Kitts and Nevis, Saint Lucia, Saint Martin, Saint Pierre and Miquelon, Saint Vincent and the Grenadines, San Marino, Serbia, Seychelles, Sint Maarten, Tokelau, Turks and Caicos Islands, U.S. Virgin Islands, Uruguay, Wallis and Futuna, and Western Sahara.

**Source:** Polaris Observatory [Internet]. Lafayette, CO: Center for Disease Analysis Foundation; 2021. https://cdafound.org/polaris

#### Persons Born in the United States Not Vaccinated as Infants Whose Parents Were Born in Regions with HBV Infection Prevalence of ≥8%

The population of persons born in the United States who were not vaccinated as infants whose parents were born in regions with HBV infection prevalence of ≥8% is at increased risk for infection. The higher underlying prevalence in this population increases the likelihood of perinatal or close contact exposures (Box 3).

#### Persons Who Use Injection Drugs or Have a History of IDU

A systematic review estimated the prevalence of HBV infection among persons who use injection drugs to be 11.8% (range = 3.5%–20%) and ever having had an infection to be 22.6% (*93*). Transmission of HBV among persons who use injection drugs might be increasing. A study of prevalence of anti-HBc in national survey data found an increase among persons who use injection drugs from 35.3% during 2001–2006 to 58.4% during 2013–2018 (*94*).

#### Persons with HIV Infection

Multiple studies with varying inclusion criteria and periods during 1986*–*2012 used prospective cohort data from the U.S. Military HIV Natural History Study (NHS) to calculate the prevalence of HBV infection among persons with HIV infection. Among patients in NHS, coinfection ranged from 3.0% to 6.0% (*95*–*97*). In a large prospective cohort study of adults with HIV infection, annual chronic HBV infection prevalence during 1996–2007 ranged from 7.8% to 8.6% (*98*).

#### MSM

Among a sample of Los Angeles County, California, residents from the National HIV Behavioral Surveillance system, 19% (95% CI = 15%–24%) of MSM had HBV infection or a history of HBV infection, and 35% of the sample were coinfected with HIV (*99*). In a survey of MSM from six U.S. metropolitan areas during 1998–2000, the prevalence of ever infection was 20.6%, and 2.3% of participants had active HBV infection; HBV infection was independently associated with a history of another STI, having more lifetime partners, ever engaging in anal intercourse, and ever using injection drugs (*100*).

#### Household, Needle-Sharing, or Sexual Contacts of Persons with Known HBV Infection

HBV is highly infectious and can survive in the environment for prolonged periods. Close (i.e., household, needle-sharing, or sexual) contacts of persons with known HBV infection are at greater risk (see Universal Screening Systematic Review and Review of Evidence Summary).

#### Persons on Dialysis, Hemodialysis, or Peritoneal Dialysis

A study during 1997–2001 of adult hemodialysis patients found an adjusted prevalence of HBV infection of 2.4% (95% CI = 2.1–2.7) (*101*). Dialysis was reported only in 3% (34 of 1,292) of 2,019 acute HBV infection cases; however, the risk for developing chronic infection was higher among persons who are immunosuppressed and undergoing dialysis than persons who are immunocompetent (*23*,*102*,*103*). *Recommendations for Preventing Transmission of Infections Among Chronic Hemodialysis Patients* includes testing recommendations for patients on hemodialysis (*104*).

#### Persons with Elevated ALT or Aspartate Aminotransferase Levels of Unknown Origin

Persons with known chronic liver disease (e.g., cirrhosis, fatty liver disease, alcoholic liver disease, or autoimmune hepatitis) are not at increased risk for HBV infection unless they have additional exposures or risk factors. However, persons with persistently elevated ALT or aspartate aminotransferase (AST) levels without a known cause should be tested for HBV infection as part of a medical evaluation of these abnormal laboratory values.

## Rationale for New Recommendations

**FIGURE 2:**
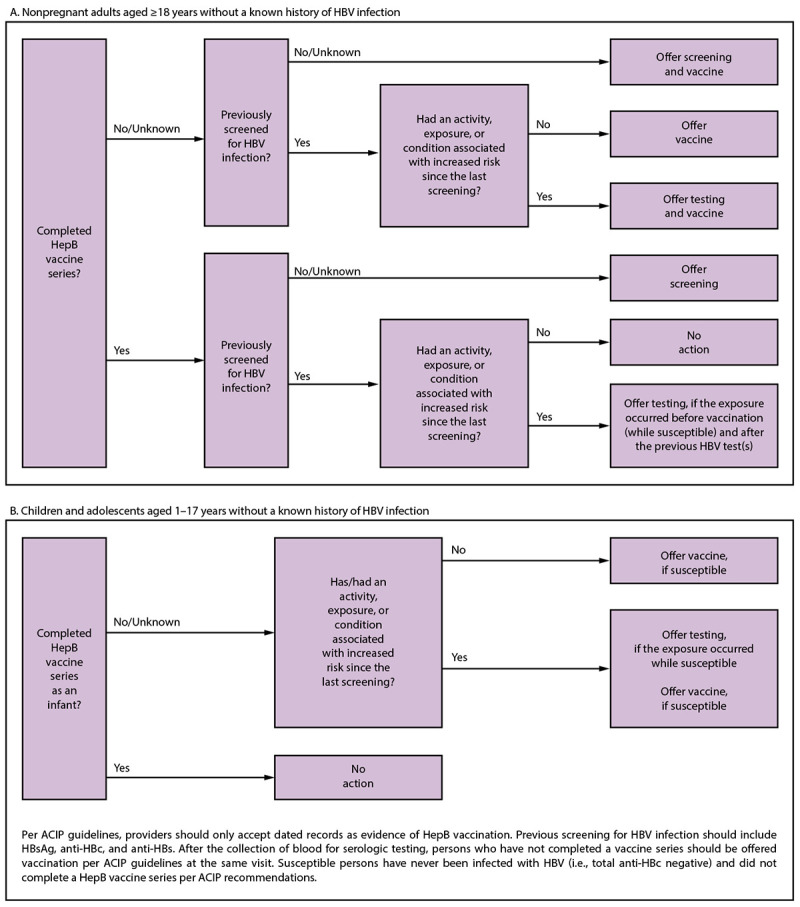
Incorporating hepatitis B virus screening and testing into a clinic workflow, by age **Abbreviations:** ACIP = Advisory Committee on Immunization Practices; anti-HBc = antibody to hepatitis B core antigen; anti-HBs = antibody to hepatitis B surface antigen; HBV = hepatitis B virus; HBsAg = hepatitis B surface antigen; HepB = hepatitis B.
- **New risk groups:** The addition of three new risk groups was based on the HBV infection prevalence cutoff of ≥1%. The selection of the three groups for which to conduct systematic reviews was based on expert judgment, and the work group recognizes other populations might also be at increased risk.

Chronic HBV infection can lead to substantial morbidity and mortality but is detectable before the development of severe liver disease using reliable and inexpensive screening tests. Routine monitoring and treatment for chronic HBV infection can reduce morbidity and mortality, supporting the importance of early detection of HBV infection. In addition, although not quantifiable, management of chronic infection through prevention efforts can prevent further transmission to others. These recommendations consider a simpler and less stigmatizing implementation strategy than previous risk-based HBV screening recommendations. The recommendations also provide guidance that is complementary to the 2022 ACIP recommendations to vaccinate all adults aged 19–59 years against HBV infection (*59*) by providing a means to establish immunity or any history of infection or the need for vaccination to protect from future infection. Specific rationales for recommendations are as follows:

- **Universal screening:** Universal screening of adults is cost-effective compared with risk-based screening and averts liver disease and death (*56*). Although a curative treatment is not yet available, early diagnosis and treatment of chronic HBV infections reduces the risk for cirrhosis, liver cancer, and death (*10*,*11*). Risk-based testing alone has not identified most persons living with chronic HBV infection and is considered inefficient for providers to implement.
- **Triple panel screening:** Using the triple panel (HBsAg, anti-HBs, and total anti-HBc) is recommended for initial screening because it can help identify persons who have an active HBV infection and could be linked to care, have resolved infection and might be susceptible to reactivation (e.g., immunosuppressed persons), are susceptible and need vaccination, or are vaccinated. When someone receives triple panel screening, any future periodic testing can use tests as appropriate (e.g., only HBsAg and anti-HBc if the patient is unvaccinated).
- **Adults aged ≥18 years:** An “all adults” recommendation was considered more feasible to implement (e.g., for integrating into electronic medical record alerts) than one among specific age groups. Considerations included the favorable economic analysis across adult age groups, similarly low vaccination rates among adult age groups, comparable epidemiology of acute and chronic infections from surveillance data among age groups, and harms of missed identification of chronic infections.
- **Children and adolescents aged <18 years:** Children and adolescents aged <18 years were not included in the universal screening recommendation because of the low prevalence of HBV infection in this age group and high levels of HepB vaccination. Children and adolescents aged <18 years who have risk factors and did not receive a complete vaccine series should be tested (Figure 2).

## HBV Screening and Testing Recommendations

In these guidelines, “screening” refers to conducting serologic testing of asymptomatic persons not known to be at increased risk for exposure to HBV. “Testing” refers to conducting serologic testing of persons with symptoms or who are identified to be at increased risk for exposure to HBV. The following evidence-based recommendations for HBV screening update and expand those issued by CDC in 2008 (*14*).

Screening is recommended for the following persons (Box 1):

- All adults aged ≥18 years at least once during a lifetime (new recommendation).
- All pregnant persons* during each pregnancy, preferably in the first trimester, regardless of vaccination status or history of testing (*15*) (see Clinical Considerations).

Testing is recommended for the following persons (Box 1):

- Everyone with a history of risk for HBV infection, regardless of age, if they might have been susceptible during the period of risk (Box 4) (Figure 2). Susceptible persons include those who have never been infected with HBV (i.e., total anti-HBc negative) and either did not complete a HepB vaccine series per ACIP recommendations or who are known vaccine nonresponders (*15*).
  BOX 4 **Persons and activities, exposures, or conditions associated with an increased risk for hepatitis B virus infection — CDC testing recommendations, 2013**
  - Infants born to pregnant persons who are hepatitis B surface antigen positive
  - Persons born in regions with hepatitis B virus (HBV) infection prevalence of ≥2%
  - U.S.-born persons not vaccinated as infants whose parents were born in regions with HBV infection prevalence of ≥8%
  - Injection drug use
  - Incarceration in a jail, prison, or other detention setting (new recommendation)
  - HIV infection
  - Hepatitis C virus infection (new recommendation)
  - Men who have sex with men
  - Sexually transmitted infections or multiple sex partners (new recommendation)
  - Household contacts of persons with known HBV infection
  - Needle-sharing or sexual contacts of persons with known HBV infection
  - Maintenance dialysis, including in-center or home hemodialysis and peritoneal dialysis
  - Elevated alanine aminotransferase or aspartate aminotransferase levels of unknown origin
  - Persons who request HBV testing (new recommendation)
- Susceptible persons, regardless of age, with ongoing risk should be tested periodically, while risk persists (Figure 2) (see Clinical Considerations).
  - Offer testing if the risk for exposure occurred after previous HBV serologic testing and while the person was susceptible.
- Anyone who requests HBV testing. These persons should receive testing, regardless of disclosure of risk, because many persons might be reluctant to disclose stigmatizing risks (new recommendation).
- Persons who have an increased risk for acquiring HBV infection, including the following:
  - Infants born to HBsAg-positive pregnant persons (*15*)
  - Persons born in regions with HBV infection prevalence of ≥2% (Box 3)
  - U.S.-born persons not vaccinated as infants whose parents were born in regions with HBV infection prevalence of ≥8% (Box 3)
  - Persons who are injecting drug users or have a history of IDU
  - Persons incarcerated or formerly incarcerated in a jail, prison, or other detention setting (new recommendation)
  - Persons with HIV infection
  - Persons with HCV infection or a past HCV infection (new recommendation)
  - Men who have sex with men
  - Persons with STIs or past STIs or multiple sex partners (new recommendation) (see Clinical Considerations)
  - Household contacts or former household contacts of persons with known HBV infection
  - Needle-sharing or sexual contacts of persons with known HBV infection
  - Persons on maintenance dialysis, including in-center or home hemodialysis and peritoneal dialysis (*104*)
  - Persons with elevated ALT or AST levels of unknown origin

Providers should follow these recommendations when offering screening and testing:

- During the initial screening, test for HBsAg, anti-HBs, and total anti-HBc (new recommendation).
  - Screening with the three tests (triple panel) can help identify persons who have an active HBV infection and could be linked to care, have resolved infection and might be susceptible to reactivation (e.g., immunosuppressed persons), are susceptible and need vaccination, or are vaccinated (Table 1). Anti-HBs of ≥10 mIU/mL is a known correlate of protection only when testing follows a complete HepB vaccine series.
- After the collection of blood for serologic testing, persons who have not completed a vaccine series should be offered vaccination per ACIP recommendations at the same visit or at an associated provider visit (*105*). Blood collection before vaccination is recommended because transient HBsAg positivity has been reported for up to 18 days after vaccination.
  - Providers do not need to wait for the serologic testing results to administer the first or next dose of vaccine.
  - Although screening can identify persons who are unvaccinated and susceptible to HBV infection, screening should not be a barrier to HepB vaccination, especially in populations that have decreased engagement with or access to health care (*59*). In settings where testing is not feasible or is refused by the patient, vaccination of persons should continue according to ACIP recommendations. Serologic testing should continue to be offered at future visits.

Additional screening might be recommended for certain populations, including blood donors, newly arrived refugees, and persons initiating cytotoxic or immunosuppressive therapy, and additional testing might be recommended for patients on hemodialysis, health care personnel, perinatally exposed infants, and persons involved in exposure events that might warrant postexposure prophylaxis and postvaccination serologic testing. Recommendations for these groups are described elsewhere (*14**,**15**,**104**,**106*–*110*). The new recommendation described in this report to include a total anti-HBc test during universal adult screening will support identification of persons with past HBV infection who should be aware of their risk for reactivation in the context of immunosuppression.

### Clinical Considerations

Frequency of periodic testing should be a shared decision between the patient and provider and based on individual risk factors, including age and immune status. For periodic testing, providers should consider using the triple panel test or AASLD’s testing strategies (e.g., anti-HBc followed by HBsAg and anti-HBs, if positive). 

Having multiple sex partners can increase the risk for exposure to HBV and other STIs; however, evidence is insufficient to specify the number of sex partners and the optimal time frame for screening to identify cases of chronic infection. Thus, clinical judgment should be used to determine risk for exposure with consideration of the number of partners, type of sex, frequency of sex, and timing of the last serologic test when recommending testing for persons with multiple sex partners.

In the interest of completing adult HBV screening, prenatal visits are an opportunity to offer the triple panel to a pregnant person and link the patient to care or vaccinate as needed. Pregnant persons with a history of appropriately timed triple panel screening and without subsequent risk for exposure to HBV (i.e., no new HBV exposures since triple panel screening) only need HBsAg screening. Testing pregnant persons known to be chronically infected or immune enables documentation of the HBsAg test result during that pregnancy to ensure timely prophylaxis for exposed infants.

Universal screening complements a robust HepB vaccination program. Documentation of HepB vaccine administration in the medical record provides verification of vaccination. Per ACIP recommendations, providers should only accept dated records as evidence of HepB vaccination (*15*). For persons who are unvaccinated or partially vaccinated (e.g., did not complete a full series), HepB vaccine should be administered immediately after collection of the blood for serologic testing. Persons with evidence of active HBV infection (i.e., HBsAg positive) or a past HBV infection (i.e., total anti-HBc positive) do not need additional vaccine doses (*15*).

### Follow-Up After HBV Testing

#### Persons with Active HBV Infection

Patients with acute infection should be counseled about their risk for developing chronic HBV infection, the risk for reactivation, and the risk for transmission to others. Treatment for acute HBV infection is not typically indicated except among patients with severe disease (*11*).

Persons who receive a diagnosis of chronic HBV infection can benefit from monitoring and counseling, including mental health support (*111*). CDC treatment guidelines have not been developed and are beyond the scope of these screening guidelines. However, AASLD has guidance for the monitoring and treatment of chronic HBV infection (*11*). Simplified guidance for primary care medical providers or other nonspecialists is available from the Hepatitis B Primary Care Workgroup (Table 2) (*112*).

**TABLE 2:** Initial medical evaluation of persons who are hepatitis B surface antigen positive

History/Examination	Patient education	Routine laboratory tests	Serology/Virology	Imaging/Staging studies
• Symptoms/signs of cirrhosis • Alcohol screening and brief intervention • Metabolic risk factors • Family history of hepatocellular carcinoma • Hepatitis A vaccination status; offer vaccine if unvaccinated	• Educate patients on how to prevent transmission to others • Identify household contacts, sex partners, or needle-sharing contacts for screening and vaccination • Recommend abstinence or limited use of alcohol* • Recommend steps to reduce risk for metabolic syndrome and fatty liver • Refer to harm reduction counseling or drug treatment services, as needed	• CBC • Comprehensive metabolic panel, including AST/ALT, total bilirubin, alkaline phosphatase, albumin, creatinine, and INR	• HBeAg/anti-HBe • HBV DNA • Anti-HAV (total or IgG) to determine need for vaccination if none documented • Anti-HCV • Anti-HDV^†^ • Anti-HIV • Other STIs (as indicated)	• Abdominal ultrasound with or without AFP^§^ • Elastography (e.g., FibroScan) or serum fibrosis assessment (e.g., APRI, FibroSure, FIB-4)

**Source:** Table adapted from Tang AS, Thornton K; Hepatitis B Primary Care Workgroup. Hepatitis B management: guidance for the primary care provider. Seattle, WA: University of Washington National Hepatitis Training Center; 2020.

**Abbreviations:** AFP = alpha fetoprotein; anti-HAV = antibody to hepatitis A virus; anti-HBe = antibody to hepatitis B e antigen; anti-HCV = antibody to hepatitis C virus; anti-HDV = antibody to hepatitis D virus; APRI = AST to platelet ratio index; AST/ALT = aspartate aminotransferase/alanine aminotransferase; CBC = complete blood count; HBeAg = hepatitis B e antigen; HBsAg = hepatitis B surface antigen; HBV = hepatitis B virus; INR = international normalized ratio; STI = sexually transmitted infection.

* More than seven alcoholic drinks/week for women and more than 14 drinks/week for men is associated with increased risk for liver disease (**Source:** Terrault NA, Lok ASF, McMahon BJ, et al. Update on prevention, diagnosis, and treatment of chronic hepatitis B: AASLD 2018 hepatitis B guidance. Hepatology 2018;67:1560–99).

^†^ **Source:** AASLD Practice Guidelines (https://www.aasld.org/practice-guidelines).

^§^ Ultrasound for hepatocellular carcinoma surveillance has higher diagnostic accuracy than AFP; therefore, AFP alone is not recommended except when ultrasound is unavailable or unaffordable (**Source:** Terrault NA, Lok ASF, McMahon BJ, et al. Update on prevention, diagnosis, and treatment of chronic hepatitis B: AASLD 2018 hepatitis B guidance. Hepatology 2018;67:1560–99).

All patients who test positive for active HBV infection should be provided information on how to prevent transmission to others (Box 5). Notification, testing, and vaccination of their household contacts or former household contacts, sex partners, and needle-sharing contacts are recommended, as appropriate. As resources allow, viral hepatitis or STI programs within local or state health departments might be available to support providers with contact tracing and notification.

BOX 5 **Prevention messages for persons with hepatitis B virus infection**

- To prevent or reduce risk for transmission to others, persons who are hepatitis B surface antigen (HBsAg) positive should take the following actions:
  - Notify their household, sexual, and needle-sharing contacts that they should be tested for markers of hepatitis B virus (HBV) infection; if susceptible, contacts should complete the hepatitis B (HepB) vaccine series
  - Use condoms to protect susceptible sex partners from acquiring HBV infection from sexual activity until the sex partners can be vaccinated and their immunity documented (condoms and other prevention methods can also reduce risks for other sexually transmitted infections
  - Cover cuts and skin lesions to prevent spread of infectious secretions or blood
  - Clean blood spills with bleach solution*
  - Refrain from donating blood, plasma, tissue, or semen
  - Refrain from sharing household articles (e.g., toothbrushes and razors) that could become contaminated with blood
  - Refrain from sharing needles, syringes, and other injection equipment
  - Dispose of blood, body fluids, and medical waste properly
- Newborns of pregnant persons who are HBsAg positive should receive the HepB vaccine and HepB immune globulin at birth and complete the HepB vaccine series according to the recommended vaccination schedule.^†^
- When seeking medical or dental care, persons who are HBsAg positive should tell those responsible for their care of their HBsAg status so they can be evaluated and managed appropriately.
  - **Source:** Rutala WA, Weber DJ; Healthcare Infection Control Practices Advisory Committee. Guideline for disinfection and sterilization in healthcare facilities, 2008. Atlanta, GA: US Department of Health and Human Services, CDC; 2008. https://stacks.cdc.gov/view/cdc/47378
    ^†^ **Source:** Schillie S, Vellozzi C, Reingold A, et al. Prevention of hepatitis B virus infection in the United States: recommendations of the Advisory Committee on Immunization Practices. MMWR Recomm Rep 2018;67:(No. RR-1):1–31.

Persons living with HBV infection have rights protected under the Americans with Disabilities Act (*113*). Persons should not be excluded from practicing in the health care field or from school, play, child care, work, or other settings because of their HBV infection (*114*,*115*).

#### Persons with Resolved (Past) HBV Infection

Patients should be counseled about their history of HBV infection and risk for reactivation. Therapies with the highest risk for reactivation include B-cell depleting agents (e.g., rituximab and ofatumumab). American Society of Clinical Oncology and AASLD guidelines have more information on therapies and conditions associated with increased risk for reactivation, as well as recommendations for treatment (*11*,*109*,*116*,*117*). Antiviral therapy for HBV infection, when initiated before immunosuppressive or cytotoxic therapy, can prevent reactivation of disease (*118*). The systematic review indicated the prevalence of resolved HBV infection (i.e., HBsAg negative and anti-HBc positive) in the general population ranges from 4.8% to 14.0% (median = 6.2%). Notification, testing, and vaccination of household, sex partners, and needle-sharing contacts of patients with HBV infection or a history of HBV infection are recommended, as appropriate.

#### Persons Who Are Susceptible to HBV Infection

Persons who are susceptible to HBV infection should be told that they have never been infected with HBV and are not protected from future infection. All persons who are susceptible to infection should be offered HepB vaccine per ACIP recommendations (*59*). Anti-HBs concentrations can wane over time among vaccine responders. For persons with a clearly documented vaccination series who test negative for anti-HBs, refer to *Prevention of Hepatitis B Virus Infection in the United States: Recommendations of the Advisory Committee on Immunization Practices* (*15*). Vaccine should be offered to persons who have initiated, but not completed, the HepB vaccine series, regardless of anti-HBs status. HepB vaccine series completion is important for long-term immunogenicity.

Persons who are susceptible, refuse vaccination, and are at increased risk for HBV infection should be periodically tested. Frequency of periodic testing should be a shared decision between the patient and provider and be based on individual risk factors and immune status.

#### Persons Who Are Fully Vaccinated Against HBV Infection

Persons are considered fully vaccinated if they have completed a HepB vaccine series and can be reassured about protection against future illness. Vaccination status should be clearly documented in the medical record. Anti-HBs concentrations can wane over time among vaccine responders (*20*). For persons with a clearly documented vaccination series who test negative for anti-HBs, refer to *Prevention of Hepatitis B Virus Infection in the United States: Recommendations of the Advisory Committee on Immunization Practices* for specific populations for whom revaccination might be recommended (e.g., patients on hemodialysis) (*15*). Revaccination or booster doses are not routinely recommended for persons who are immunocompetent (*15*).

#### Persons with Isolated Core Antibody

Persons with isolated anti-HBc should have their immune status and risk history considered before deciding next steps. Links to performance characteristics on all FDA-approved total anti-HBc assays are available (Supplementary Table 21, https://stacks.cdc.gov/view/cdc/124432). The specificity of total anti-HBc tests is 99.8% (*119*,*120*). However, if a person does not have risk factors, the result might be a false positive; repeat testing with the same assay is warranted to confirm the results (*121*). A false-positive isolated core antibody result means the person is susceptible and should be offered HepB vaccine per current ACIP recommendations (*105*).

A 2001–2018 national survey found the prevalence of isolated positive anti-HBc to be 0.8% (approximately 2.1 million persons) (*122*). Among patients exposed to HBV, an isolated positive anti-HBc result might be the result of loss of anti-HBs after past resolved infection, occult infection (i.e., HBsAg is negative, but HBV DNA is positive), being in the window period before appearance of anti-HBs, or an HBsAg mutant infection (i.e., an infection that is not picked up by an HBsAg test unable to detect mutants). Patients who are immunosuppressed should be considered at risk for HBV reactivation, and HBV DNA testing is recommended to assess for occult infection (*11*). Among infants, an isolated anti-HBc result might be a consequence of passive placental transfer from an HBsAg-positive mother, which is why testing for anti-HBc is not indicated before age 24 months (*15*).

### Patient Education

Patient education should be conducted in a culturally sensitive, nonstigmatizing manner in the patient’s primary language (both written and oral whenever possible). Bilingual, bicultural, and medically trained interpreters should be used when indicated.

### Reporting

Acute and chronic cases of HBV infection should be reported to the appropriate state or local health jurisdiction in accordance with requirements. The Council of State and Territorial Epidemiologists publishes case definitions for the classification of reportable cases of HBV infection (*123*,*124*). CDC has updated guidance for health departments on viral hepatitis surveillance and case management (*125*).

## Recommendations and Guidance from Non-CDC Sources

USPSTF, AASLD, and ACP also have published HBV screening recommendations. The 2021 USPSTF systematic review found that no study directly evaluated the effects of screening for HBV infection on clinical outcomes and that risk-based screening strategies identify nearly all patients with HBV infection (*126*). USPSTF recommends screening adolescents and adults at increased risk for HBV infection with HBsAg tests. Although the work group did not identify any studies directly comparing the effects of universal screening versus risk-based screening, the cost-effectiveness analysis, indirect evidence on the effects of screening, practicality of implementing guidelines, public health benefits, and subject matter expertise were considered. The work group concluded that the benefits of universal screening outweighed the costs.

AASLD also recommends screening persons at increased risk for infection; however, this guidance primarily is based on previous CDC recommendations. AASLD guidance differs from CDC guidance by recommending screening of unvaccinated persons with diabetes aged 19–49 years, travelers to countries with intermediate or high prevalence of HBV infection, and residents and staff of facilities for developmentally disabled persons (*11*). CDC recommends universal adult screening, but not periodic testing, for these groups. AASLD also only recommends anti-HBc testing for certain groups (*11*).

In their best practice advice, ACP and CDC recommend testing persons at increased risk for HBV infection with tests for HBsAg, total anti-HBc, and anti-HBs (*121*). The ACP best practice risk groups align with current testing recommendations except that ACP omits persons with a history of STIs or multiple sex partners (Box 4).

CDC screening guidelines were developed independently from the ACIP recommendations for HepB vaccination. The 2018 ACIP recommendations also include recommendations for serologic testing (*15*). CDC’s screening and testing guidelines cover all persons recommended for serologic testing per ACIP and expand beyond that list (Box 1). When considering prevaccination testing or testing for HBV infection, CDC recommends testing that follows a universal testing approach (Figure 2).

The work group did not evaluate clinical guidance for patients after screening. The most recent expert guidance to reduce the risk for liver damage is that patients with HBV infection should be vaccinated against hepatitis A virus (if not already immune) (*112*); screened for HIV, HCV, and hepatitis D virus (HDV) (*112*); and assessed for alcohol risk factors, such as with the alcohol screening and brief intervention (*127*). Depending on the likely route of transmission, the patient might benefit from STI screening, drug treatment, or harm-reduction counseling. A full list of recommended steps for examination, education, laboratory, serology, and imaging is provided (Table 2).

Additional screening might be recommended for blood donors, newly arrived refugees, and persons initiating cytotoxic or immunosuppressive and additional testing might be recommended for patients on hemodialysis, health care personnel, perinatally exposed infants, and persons involved in exposure events who might warrant postexposure prophylaxis and postvaccination serologic testing. These recommendations are described elsewhere (*14**,**15**,**104*,*106*–*110*). Providers should follow the most conservative approach when recommendations differ.

## Future Directions

CDC will review these recommendations as new treatments, tests, epidemiology, HepB vaccination rates, and experience gained from implementation of these recommendations become available; recommendations will be revised as needed. The work group did not conduct a systematic review to reassess any of the groups at increased risk for HBV infection from the 2008 guidelines; future recommendations might modify the groups recommended for periodic testing. Additional data on the ideal frequency of periodic testing is needed. Continued collaboration with laboratories to bundle the three HBV tests (HBsAg, anti-HBs, and anti-HBc) would facilitate ordering the tests together as a triple panel. In addition, reporting a triple panel summary result will aid providers in correctly interpreting results. Finally, a better understanding of the prevalence of HDV in the United States is needed to inform recommendations for HDV screening among persons with HBV infection.

## Conclusion

Universal screening of adults for HBV infection is cost-effective compared with risk-based screening and averts liver disease and death (*56*). Although a curative treatment is not yet available, early diagnosis and treatment of chronic HBV infections reduces the risk for cirrhosis, liver cancer, and death (*10*,*11*). Risk-based testing alone has not identified most persons living with chronic HBV infection and is inefficient for providers to implement. Along with vaccination strategies, universal screening of adults and appropriate testing of persons at increased risk for HBV infection will improve health outcomes, reduce the prevalence of HBV infection in the United States, and advance viral hepatitis elimination goals.

## Footnotes

* Pregnant person refers to anyone who can give birth, regardless of gender, because these recommendations are inclusive of persons of any gender (https://uspreventiveservicestaskforce.org/uspstf/about-uspstf/methods-and-processes).
