# *Erratum:* Vol. 72, No. RR-1

**DOI:** 10.15585/mmwr.mm7232a5

**Published:** 2023-08-11

In the Recommendation and Report, “Screening and Testing for Hepatitis B Virus Infection: CDC Recommendations — United States, 2023,” on page 20, in line 4 under Contributors, the affiliation for Elisa Choi should have read, “Harvard **Medical School** and American College of Physicians.”

